# Choroidal Neovascularization and Toxic Optic Neuropathy Secondary to Tobacco Use: A Case Report

**DOI:** 10.7759/cureus.11493

**Published:** 2020-11-15

**Authors:** Yasmin Islam, Gibran Khurshid

**Affiliations:** 1 Ophthalmology, University of Florida, Gainesville, USA

**Keywords:** optic neuropathy, tobacco use disorder, alcohol use disorder, choroidal neovascularization

## Abstract

Tobacco and alcohol dependence are known to cause choroidal neovascularization and toxic optic neuropathy, although these typically occur in isolation. In this case report, we describe a 54-year-old male who presented with a juxtafoveal choroidal neovascular membrane (CNVM) in the left eye. Over the course of the next two years, his vision worsened significantly in both eyes, and he developed decreased color vision and paracentral scotomas. Impaired photoreceptor response was detected on full-field electroretinography in both eyes. MRI of the brain and orbits was normal, and laboratory tests for optic neuropathy were within normal limits, except for highly elevated cotinine and nicotine levels. He was in the habit of chewing tobacco nearly constantly, and he admitted to drinking 15-20 alcoholic beverages per week. He was diagnosed with choroidal neovascularization and optic atrophy due to tobacco and alcohol overuse. The effects of tobacco and alcohol use on the health of the choroidal vasculature and optic nerve are discussed in the article.

## Introduction

Tobacco and alcohol dependence have adverse effects on the choroidal and retinal vasculature, as well as the function of the optic nerve. Despite multiple public health campaigns to decrease rates of tobacco and alcohol use, dependence on these substances remain high. Additionally, tobacco addiction correlates with an increased risk of alcohol use disorder; patients addicted to tobacco are 2.1 times more likely to use alcohol compared to patients without tobacco dependence [1]. Using alcohol with tobacco can potentiate each other’s ill effects on ocular functioning [2]. In this case report, we describe a unique patient with both choroidal neovascularization and toxic optic neuropathy incited by overuse of chewing tobacco and alcohol.

## Case presentation

**Figure 1:**
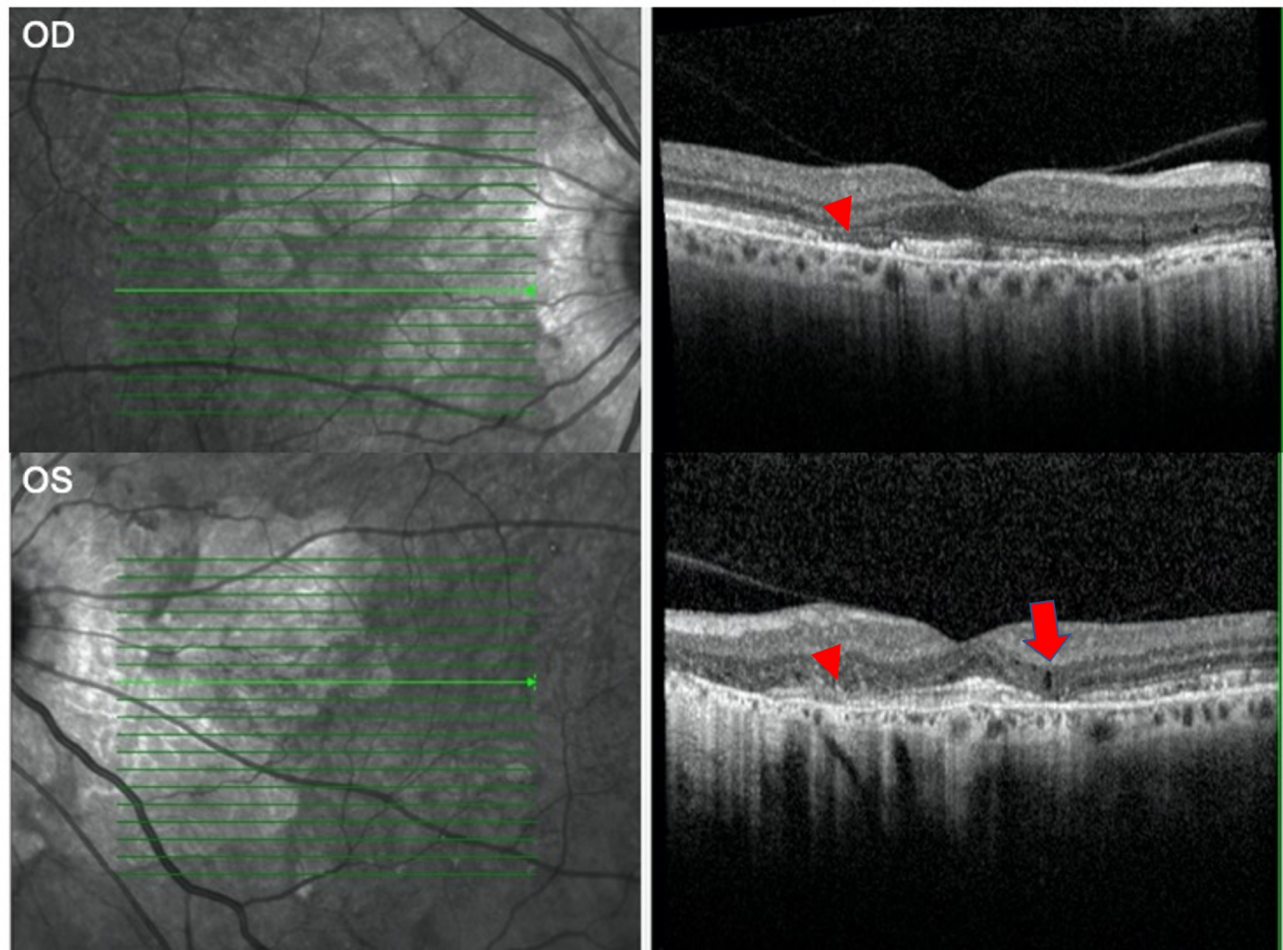
Macular optical coherence tomography Macular optical coherence tomography scan of the right eye (OD, top) and left eye (OS, bottom) at presentation. There was outer retinal atrophy and loss of the inner segment/outer segment junction in both eyes (arrowheads), as well as a small amount of intraretinal fluid in the left eye (arrow)

A 54-year-old male with a past ocular history of angioid streaks and peripapillary geographic atrophy of both eyes presented for the treatment of a juxtafoveal choroidal neovascular membrane (CNVM) in the left eye. The CNVM had developed six months prior to his presentation to the clinic. He had been treated by an outside retina surgeon with two injections of bevacizumab, an anti-vascular endothelial growth factor agent that is commonly used to combat CNVM. His past medical history was significant for obstructive sleep apnea, osteoarthritis, and benign prostatic hyperplasia. Visual acuity was 20/20 in the right eye and 20/200 in the left eye, and he had an afferent pupillary defect in the left eye. Dilated fundus exam demonstrated geographic atrophy and angioid streaks in both eyes, as well as a CNVM in the left eye. Macular optical coherence tomography demonstrated retinal atrophy in both eyes, loss of inner segment/outer segment junction in both eyes, and a small amount of intraretinal fluid in the left eye (Figure 1). After discussing with the patient, the decision was made to keep him under observation for the long term. 

After two years of stability, the patient started to complain of glare and haloes, which correlated with his worsening cataract in the left eye. He underwent uncomplicated cataract extraction with intraocular lens implantation in the left eye. His visual acuity in the left eye improved to 20/40. Three months after cataract surgery, his vision worsened to 20/60 in the right eye and 20/80 in the left. He also started to complain of worsening scotomas in the left eye, decreased color vision, and intractable headaches. A month later, he developed new flashes of light and floaters in both eyes. Multiple dilated fundus exams demonstrated stability to his initial exam. Given his worsening symptoms, a full-field electroretinogram was performed, which demonstrated extinguished scotopic and photopic responses in both eyes, indicating photoreceptor damage. Over the next few months, his vision worsened to 20/400 in both eyes.

A systemic investigation into the underlying cause of his CNVM and optic neuropathy was pursued. Laboratory testing demonstrated an elevated nicotine level of 24.7 ng/mL (reference interval: <2 ng/mL) and elevated cotinine level of 280.2 ng/mL (reference interval: <20 ng/mL). The remainder of a toxicology screen was negative. Vitamin B12, folate, thyroid-stimulating hormone, antinuclear antibody screen, complete blood count, and complete metabolic panel were all within normal limits (Table 1). MRI of the brain and orbits did not demonstrate any abnormalities. During clinic visits, he was consistently noticed to be chewing tobacco; he admitted to over 40 years of frequent chewing-tobacco use. He reported that he chewed approximately 70 grams of snuff daily. His preferred brand contains 3.8 mg/g of nicotine, resulting in approximately 266 grams of daily nicotine exposure. He also reported heavy alcohol use, with two drinks per day during the weekdays, and two to four drinks of hard liquor daily over the weekend. Ultimately, he was diagnosed with toxic optic neuropathy and choroidal neovascularization due to excessive tobacco and alcohol use. He was counseled to decrease his use of toxic substances, and he is currently working with his primary care physician on tobacco and alcohol use reduction.

**Table 1:** Laboratory studies Selected laboratory results demonstrating elevated nicotine and cotinine levels, indicating heavy tobacco use. All other tests, including a complete metabolic panel, complete blood count, and lipid panel, were within normal limits

Laboratory testing		
Test	Results	Reference interval
Nicotine	24.7 ng/mL	<2.0 ng/mL
Cotinine	280.2 ng/mL	<20.0 ng/mL
Creatinine	1.20 mg/dL	0.60-1.30 mg/dL
Hemoglobin A1c	5.4%	4.0-6.0%
Sedimentation rate	6 mm/h	<20 mm/h
Antinuclear antibody screen	Negative	Negative
Thyroid-stimulating hormone	2.33 mIU/L	0.4-4.50 mIU/L
Folate	>24.0 ng/mL	>5.4 ng/mL
Vitamin B12	474 pg/mL	200-1,100 pg/mL
Hemoglobin	14.4 g/dL	13.0-16.5 g/dL

## Discussion

Excessive tobacco use has been associated with both toxic optic neuropathy and CNVM. These toxic effects are magnified when coupled with excessive alcohol use. While the toxic effects of alcohol and tobacco use have been well described in the literature [2,3], to our knowledge, this is the first case demonstrating both choroidal neovascularization and optic neuropathy in the same patient from excessive substance use. A Pubmed search for all English-language articles without date restrictions for (“choroidal neovascularization” OR “choroidal neovascular membrane”) AND (“optic atrophy” OR “optic neuropathy”) AND “tobacco” AND “alcohol” did not yield any other similar cases in the literature.

While excessive substance use can cause both toxicity to the optic nerve and choroidal vasculature, this damage occurs via different mechanisms. CNVMs occur when abnormal choroidal vasculature breaks through to the neurosensory retina; subsequent hemorrhage and retinal edema often result in vision loss. Choroidal neovascularization is promoted via nicotine-induced angiogenesis, a finding that is compounded with concomitant alcohol use. Heeschen et al. first described nicotine use promoting angiogenesis throughout the body in 2001 through the mediation of nicotinic acetylcholine receptors on vascular endothelial cells [4]. Similar to its effects on systemic vascular endothelium, nicotine significantly affects the vasculature of the eye by inducing cellular hypoxia, thereby promoting CNVM formation and growth. Nicotine has been demonstrated to increase vascularity of CNVMs, likely through potentiating the effects of platelet-derived growth factor on the choroidal smooth muscle cells. This results in the growth of these cells, thus increasing the size of CNVM [5]. The adverse effects of nicotine on the choroidal vasculature are also mediated through the upregulation of vascular endothelial growth factors in nicotine use. In mice, the concomitant use of alcohol and tobacco increases the risk of CNVM formation, indicating the synergistic effects these two substances have on the formation of pathology in the choroidal vasculature [2].

On the other hand, the pathophysiology of tobacco and alcohol causing toxic optic neuropathy is less understood. However, both are thought to damage the respiratory cycle of the mitochondria [3]. Tobacco use has been associated with changes in mitochondrial morphology, resulting in a decrease in ciliary activity and resultant cell death [6]. Additionally, both tobacco and alcohol impair vascular circulation systemically, including decreasing blood flow to the optic nerve with chronic overuse [7]. As in our patient, the visual acuity can often drop to as low as 20/400 in combined tobacco-alcohol toxic optic neuropathy. When combined with choroidal neovascularization, as in our patient, vision loss can be profound.

While decreased vision from chronic misuse of tobacco and alcohol can be permanent, a reversal can be achieved with early interventions [3]. This reinforces the important role that ophthalmologists can play in counseling patients with substance use disorders about the need to seek appropriate treatment for their addictions. Due to the societal stigma against addiction, patients can be reluctant to admit the extent of their substance use. As in this case, obtaining serum levels of cotinine can be helpful in elucidating the cause of vision loss and appropriately counseling patients on cessation.

## Conclusions

In conclusion, heavy nicotine use, when combined with excessive alcohol use, can have toxic effects on both the choroidal vasculature and optic nerve. These can lead to profound and rapid vision loss. In this case report, we discussed a unique patient with both choroidal neovascularization and toxic optic neuropathy caused by the overuse of tobacco and alcohol. Appropriate counseling and cessation of substance use can potentially reverse or diminish the effects of these toxic materials.
